# Coexistence of submandibular epithelioid angiosarcoma and papillary thyroid carcinoma: A case report

**DOI:** 10.1097/MD.0000000000029341

**Published:** 2022-06-30

**Authors:** Yi-Ting Liu, Jing Wang, Yan-Xia Sui, Dong-Li Zhao

**Affiliations:** a Department of Oncological Radiotherapy, First Affiliated Hospital of Xi’an Jiaotong University, Xi’an, Shaanxi Province, China; b Department of Medical Oncology, Yan’an University Affiliated Hospital, Yan’an, Shaanxi Province, China; c Department of Endocrinology, Yan’an University Affiliated Hospital, Yan’an, Shaanxi Province, China; d Department of Pathology, First Affiliated Hospital of Xi’an Jiaotong University, Xi’an, Shaanxi Province, China.

**Keywords:** angiosarcoma, epithelioid angiosarcoma, papillary thyroid carcinoma

## Abstract

Reports on the coexistence of epithelioid angiosarcoma (EA) and papillary thyroid carcinoma (PTC) are rare. Over the past 50 years, only 2 cases of coexistence of EA and PTC have been reported in English literature. Therefore, we report a rare case of coexistence of EA and PTC treated with surgery and adjuvant radiation therapy.

**Patient concerns:**

A 64-year-old man visited our hospital with a painless mass in the left submandibular gland, with poor mobility.

**Diagnosis:**

Neck ultrasonography revealed nodules in the left submandibular gland and multiple cystic-solid mixed nodules in the left thyroid gland. Pathological findings revealed coexistence of EA in the left submandibular gland area and PTC in the left thyroid gland.

**Interventions:**

The patient underwent resection of the left submandibular gland, deep maxillofacial tumor, total thyroidectomy, left neck I, II, III, and VI regional lymph node dissection, and recurrent laryngeal nerve exploration under general anesthesia. Two months postoperatively, the patient also received adjuvant radiation therapy in the local and adjacent areas, with 4MV-X IMRT DT50GY at 2Gy/day 25 fractions.

**Outcomes:**

The follow-up period was 37 months. The patient recovered well without focal neurological deficits, local recurrence, or distant metastasis after surgery, except for grade I skin reaction after adjuvant radiation therapy.

**Conclusions:**

This is a rare case report of the coexistence of EA in the left submandibular gland and PTC in the left thyroid gland. Although multiple examinations were used, precise preoperative diagnosis was challenging owing to the coexistence of EA and PTC. Surgery and radiotherapy were effective treatments for the coexistence of EA and PTC in this case.

## 1. Introduction

Angiosarcoma is a rare and highly malignant tumor of endothelial origin. Epithelioid angiosarcoma (EA) is a rare subtype characterized by the epithelioid appearance of tumor cells. It is a highly aggressive endothelial malignant tumor that usually occurs in the deep soft tissues of the extremities; however, it can also attack other organs, including the adrenal gland, thyroid, skin, and bones.^[1]^ Papillary thyroid carcinoma (PTC) is the most common malignant tumor derived from follicular cells of the thyroid gland. In middle-aged women, the degree of malignancy is low, and the prognosis is good. The 10-year survival rate is over 90%.^[2]^ However, it is extremely rare of EA and PTC to co-exist in the salivary gland. The present case report describes submandibular EA coexisting with PTC without any history of radiation exposure and a family history of salivary gland and thyroid neoplasms. It helps investigate the occurrence, development, diagnosis, and treatment of EA and whether it is associated with thyroid neoplasms.

## 2. Case report

A 64-year-old man was admitted to our hospital with a left submandibular mass, with poor mobility, no tenderness, nausea, vomiting, dizziness, and other symptoms. He did not visit a doctor after the onset of symptoms and self-administered an “antiinflammatory drug” treatment (the specific drug and dosage were not known). However, the mass size did not decrease significantly. Three months later, the patient visited Surgery Clinic of Otolaryngology Head and Neck. The outpatient doctor performed releveant physical examinations and the results revealed a mass approximately 2 cm in diameter that could be palpated under the left side of the jaw, with a hard texture, rough surface, and poor mobility. The submandibular region on the right side of the jaw was normal. The left lobe of the thyroid was enlarged with multiple nodules; the largest nodule was approximately 3 × 3 cm, and no swollen lymph nodes were detected. The patient also underwent a neck ultrasound, which identified a left submandibular gland nodule. In the middle section of the left lobe in the thyroid gland, a mixed solid and cystic echo, 2.1 cm × 3.1 cm × 3.8 cm in size, had an irregular shape with an unclear boundary. The internal echo showed a strong striated echo and chip-like dark area. There was a hypoechoic nodule in the lower pole, 0.6 cm × 0.7 cm × 0.7 cm in size, with an irregular shape and unclear boundary, and uneven internal echo distribution. The presence of a stronge echo with acoustic shadowing was measured within the nodule. Additionally, variably sized cystic nodules and mixed cystic and solid nodules were detected in the right lobe of the thyroid gland. The patient was admitted to the Department of Surgery Clinic of the Otolaryngology Head and Neck for further surgery. After ruling out surgical contraindications, he underwent left submandibular gland resection, deep maxillofacial tumor resection, total thyroidectomy, left neck I, II, III, and VI regional lymph node dissection, and recurrent laryngeal nerve exploration under general anesthesia.

**Figures 1.**
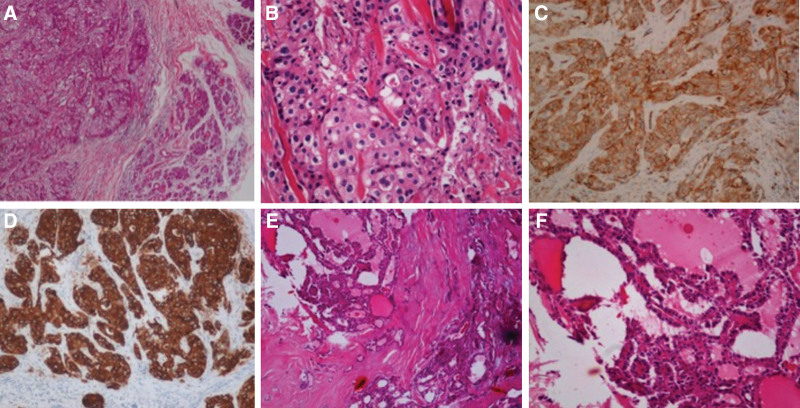
A–F: Pathological findings of submandibular gland and left thyroid gland.

The pathological tissues of the submandibular and left thyroid glands were harvested and examined. The frozen pathological results showed a submandibular gland epithelioid malignant tumor and PTC in the left thyroid gland. Immunohistochemical staining showed: CD31 (+), cytokeratin (CK) (+), epithelial membrane antigen (EMA) (+), Vim (focal +), CD34 (–), S100 (–), Tg (–), TTF1 (–), and Ki67 (+30%). Hematoxylin-eosin (HE) staining of the submandibular gland tumor showed that the tumor cells diffused into sheets or were arranged in nests with invasive growth (Fig. 1A). The tumor cells were epithelioid with obvious nuclear atypia (Fig. 1B). Immunohistochemical staining for vascular tumor-associated marker CD31 was diffusely positive (Fig. 1C), and the epithelial marker CK (Fig. 1D) and EMA were diffusely positive. HE staining of the thyroid tumor cells was arranged in a papillary pattern (Fig. 1E). The cancer cells were aligned overlapping and crowded (Fig. 1F). Based on these pathological findings, the patient was diagnosed with EA of the left submandibular gland and PTC of the left thyroid gland.

Two months postoperatively, the patient recovered well and was admitted to the radiotherapy department for adjuvant radiation therapy in the local and adjacent areas, with 4MV-X IMRT DT50GY at 2Gy/day 25 fractions. The patient was well, and only a grade I skin reaction was reported. Subsequently, the patient was followed up regularly for a period of 37 months, and no local recurrence or distant metastasis was detected.

## 3. Discussion

Angiosarcoma is a rare and highly malignant tumor of the soft tissue originating from the vascular endothelium, which accounts for <1% of all sarcomas and can occur throughout the body. Approximately 50%–52% of angiosarcoma occur in the head and neck.^[3]^ Most angiosarcoma occur in the cortex (soft tegument areas) and superficial soft tissue. However, <1% of such tumor occur in the oral and salivary glands.^[4]^

EA is a rare subtype of angiosarcoma characterized by the epithelioid appearance of tumor cells. It is a highly aggressive malignant tumor of the endothelial cells, with lymph node and solid organ metastases occurring at an early stage. Most EAs are soft tissue sarcomas,^[1]^ which usually occur in the deep soft tissues of the extremities, but also involve other major tissue organs, including the thyroid, skin, adrenal glands, and bones.

PTC is the most common malignant tumor derived from thyroid follicular cells and accounting for about 60–70% of thyroid cancers. It usually occurs in middle-aged women aged 40-50 years with a low degree of malignancy and good prognosis, with a 10-year survival rate of over 90%.^[2]^ In 2014, Mehmet Kefeli Ozgur Mete reported similar cases of EA and PTC.^[5]^

EA can occur at any age, but it is most commonly detected in middle-aged men. Clinical investigations have found that most patients do not manifest obvious symptoms, except for the symptoms of pain, fatigue, weight loss, anemia, and local compression symptoms in the late-onset stage.^[4]^ Bone invasion can cause pain in an appropriate area.^[6]^ Primary thyroid cancer can compress the trachea, leading to dyspnea,^[7]^ and lung metastasis can result in hemoptysis.^[8]^ PTC can also occur at any age; however, it is mostly found in middle-aged and elderly women. It generally presents with no obvious clinical symptoms. A painless mass in the neck can usually be palpated, and thyroid nodules are often observed during physical examination, as confirmed by needle biopsy. Thyroid-stimulating hormone levels are closely associated with PTC recurrence, metastasis, and cancer-related death, especially in high-risk PTC patients.^[9]^ In addition, Huy Gia Vuong^[10]^ published a meta-analysis on the prognosis of PTC patients with BRAF and TERT promoter mutations in clinical endocrinology in March 2017. The results showed that PTCs with concurrent BRAF and TERT promoter mutations were associated with tumor invasiveness and that BRAF or TERT promoter mutations were less aggressive. These mutation results will help to better evaluate the prognosis patients with PTC.

EA consist of large, slightly pleomorphic, round to polygonal epithelioid cells with central-to-eccentric nuclei and prominent nucleoli in the nuclei. Neoplastic cells diffuse into a sheet and can also be irregularly tubular or nested. Irregular shapes of vascular lumens of different sizes can be observed between cells, which are lined with atypical endothelial cells. CD31 is the most sensitive vascular marker for angiosarcoma diagnosis.^[6]^ More than 90% of CD31 expression is observed in angiosarcoma, but <1% of CD31 is positive for cancer cells.^[1]^ Therefore, CD31 is considered to provide a relatively high sensitivity and specificity index.^[11]^ The rate of CD34 positivity is 40%–100%, which is easy to stain in the obvious angiogenic region. However, the specificity of this marker is low, and the rate at which CD34 tests are positive for poorly differentiated lesions is limited.^[1]^

EA is aggressive and characterized by epithelioid tumor cells that can mimic many other types of epithelioid malignancies.^[1]^ It is important to differentiate EA from metastatic carcinoma and epithelioid hemangio endothelioma (EHE).^[12]^ EHE is highly invasive and its standard treatment is extensive surgical resection with adjunctive radiotherapy and/or chemotherapy. Both EA and metastatic carcinoma express keratin and epithelial membrane antigen. However, the most common metastatic lesions are negative for CD31 and V III RA, and EA is diagnosed as positive for CD31 and V III RA.^[12]^ EHE usually occurs in young patients, and the degree of malignancy varies between angiosarcoma and hemangioma. Nuclear polymorphism of EHE cells can be observed by HE staining, but the degree is less than that of EA. Surgery is the primary treatment for EHE, and prostheses and autogenous revascularization can be used.^[13]^

Currently, there are only a few reports on EA treatment. Early surgery remains the primary treatment, supplemented by postoperative radiotherapy and/or chemotherapy.^[14]^ For patients with an unfavorable prognosis due to recurrence and metastasis after surgery and chemoradiotherapy, everolimus is an oral rapamycin derivative that exerts anticancer effects by forming mTOR complex 1 (mTORC1) with FK506 Blinding Protein 12KD (FKBP12). Two patients who failed to recover after active surgery, radiotherapy, or chemotherapy were administered with allolimus (10 mg/day). Both patients experienced pain relief and improved quality of life 3 days after the oral administration of everolimus. Ten days later, PET/CT imaging of patient II showed a significant decrease in the tumor volume. The SUVmax decreased from 17.8 to 2.7. The progression free survival of the patients were nearly 12.0 and 6.0 months, and the overall survival was approximately 18.0 and 10.0 months, as reported by Shi-Long Zhang.^[14]^

Surgical resection, postoperative treatment, and thyroid-stimulating hormone (TSH) inhibition therapy are the main treatments for PTC. Currently, the choice of surgery and the extent of thyroidectomy remain controversial. J. Kenneth Byrd^[15]^ believed that PTC lesions >1 cm were suitable for total thyroidectomy, whereas lobectomy and isthmus resection were suitable for single lesions > 1 cm without adverse prognostic factors. Paolo Miccoli^[16]^ published a review in 2017; however, the relationship between the extent of thyroidectomy and long-term survival is not well established, and PTC focuses primarily on long-term survival and recurrence. According to the available literature, no single surgical strategy is appropriate for all patients. When the decisions about the extent of thyroidectomy should be carefully combined with preoperative, intraoperative, and postoperative prognosis and risk assessment, and the risk redenction of recurrence as far as is acceptable. Yasuhiro^[17]^ followed-up 5897 PTC patients with a median follow-up of 177 months. The results showed that in patients with primary lesions > 4 cm, age ≥ 45 years, lymph node metastasis, or distant metastasis, ^131^I should be used to remove residual cancer tissue. Thyroxine should be administered to prevent TSH levels from being below the normal or lower limit, or even undetectable. However, because oral thyroxine can cause TSH fluctuations and its long-term use can affect the quality of life of patients, it is not recommended for long-term prevention.^[15]^

## 4. Conclusions

The coexistence of EA in the left submandibular gland and PTC in the left thyroid gland is very rare. Currently, only 2 cases of concurrent of EA and PTC have been reported in the English literature over the past 50 years. Precise preoperative diagnosis remains challenging, despite the use of multiple diagnostic techniques. Surgery is the most common modality for the coexistence of EA and PTC, followed by radiotherapy and/or chemotherapy, which helps prolong survival time in patients with such conditions.

### Author contributions

YTL analyzed and interpreted the patient data regarding the epithelioid angiosarcoma and papillary thyroid carcinoma. JW drafted the manuscript and contributed to the literature review. YXS performed the histological examination of the tumors. DLZ revised the manuscript critically for important intellectual content. All authors have read and approved of the manuscript.

Patient consent for publication: Written consent was obtained from the patient to publish this case report and any accompanying images.
